# SpRY Cas9 Can Utilize a Variety of Protospacer Adjacent Motif Site Sequences To Edit the Candida albicans Genome

**DOI:** 10.1128/mSphere.00303-21

**Published:** 2021-05-19

**Authors:** Ben A. Evans, Douglas A. Bernstein

**Affiliations:** a Department of Biology, Ball State University, Muncie, Indiana, USA; University of Georgia

**Keywords:** CRISPR, *Candida albicans*, PAM site, genome editing

## Abstract

Candida albicans is a human fungal pathogen capable of causing life-threatening infections. The ability to edit the C. albicans genome using CRISPR/Cas9 is an important tool investigators can leverage in their search for novel therapeutic targets. However, wild-type Cas9 requires an NGG protospacer adjacent motif (PAM), leaving many AT-rich regions of DNA inaccessible. A recently described near-PAMless CRISPR system that utilizes the SpRY Cas9 variant can target non-NGG PAM sequences. Using this system as a model, we developed C. albicans CRISPR/SpRY. We tested our system by mutating C. albicans *ADE2* and show that CRISPR/SpRY can utilize non-NGG PAM sequences in C. albicans. Our CRISPR/SpRY system will allow researchers to efficiently modify C. albicans DNA that lacks NGG PAM sequences.

**IMPORTANCE** Genetic modification of the human fungal pathogen Candida albicans allows us to better understand how fungi differ from humans at the molecular level and play essential roles in the development of therapeutics. CRISPR/Cas9-mediated genome editing systems can be used to introduce site-specific mutations to C. albicans. However, wild-type Cas9 is limited by the requirement of an NGG PAM site. CRISPR/SpRY targets a variety of different PAM sequences. We modified the C. albicans CRISPR/Cas9 system using the CRISPR/SpRY as a guide. We tested CRISPR/SpRY on C. albicans *ADE2* and show that our SpRY system can facilitate genome editing independent of an NGG PAM sequence, thus allowing the investigator to target AT-rich sequences. Our system will potentially enable mutation of the 125 C. albicans genes which have been previously untargetable with CRISPR/Cas9. Additionally, our system will allow for precise targeting of many genomic locations that lack NGG PAM sites.

## OBSERVATION

Candida albicans is the most prevalent human fungal pathogen. Infections by C. albicans range in severity from uncomfortable rashes to life-threatening invasive candidiasis of which up to 50% of cases are lethal (1–3). Immunocompromised individuals are at an increased risk for invasive candidiasis, and the increased prevalence of antifungal-resistant strains can complicate treatment (4). Further characterization of C. albicans biology is necessary to identify novel therapeutic targets.

Genetic engineering is a powerful tool for elucidating the function of genes and as such can help identify potential therapeutic targets. However, C. albicans is diploid, can undergo chromosome loss and homozygosis, cannot maintain plasmids, and does not have a known meiotic cycle (5–7). Together, these factors reduce the efficiency of traditional genetic engineering techniques in C. albicans.

The development of CRISPR-mediated genome editing for C. albicans has allowed for more efficient manipulation of the genome (8–10). C. albicans CRISPR utilizes a yeast codon-optimized Cas9 that is targeted by a guide RNA to a cDNA sequence to site specifically introduce a double-strand break (8, 11). The break is repaired, and mutations are introduced by homologous recombination with a cotransformed repair template (8). Some methods require that *CAS9*, the guide RNA, and a selection marker be incorporated into the C. albicans genome, while others transiently express Cas9 and guide RNA, eliminating the need for incorporation of the CRISPR cassette (8, 9).

Although CRISPR/Cas9 has increased the efficiency by which investigators can change the C. albicans genome, it is limited by the requirement for a 5′-NGG-3′ protospacer adjacent motif (PAM) immediately downstream of the guide sequence (12). In bacteria, this PAM site requirement functions to distinguish self from nonself so bacteria do not cleave their own genome (13). However, this requirement during genome editing leaves DNA lacking NGG sequences unavailable for targeting with traditional Cas9. A recent Cas9 variant, designated SpRY, utilizes alternative PAM site sequences in human cells (14). CRISPR/SpRY enables mutation at NRN sequences, where R = A or G, more efficiently than at NYN sequences, where Y = C or T. Building upon this technology, we have developed a CRISPR/SpRY-mediated genome editing system for C. albicans by introducing the mutations from the SpRY variant into the yeast codon-optimized Cas9. We find that CRISPR/SpRY can utilize alternative PAM sites in C. albicans.

### Generation of SpRY Cas9 plasmid vectors.

Mutations that encoded the following amino acid changes were introduced via gene synthesis into yeast codon-optimized *CAS9*; A61R, L1111R, D1135L, S1136W, G1218K, E1219Q, N1317R, A1322R, R1333P, R1335Q, and T1337R (GenScript, Piscataway, NJ). Yeast codon-optimized *CAS9* was removed from plasmid vectors pV1093, pV1393, and pV1524 via restriction digestion with SmaI and ApaI. The mutated construct was then ligated into cut pV1093, pV1393, and pV1524. Plasmids were verified via sequencing. The resulting plasmids are designated pV1093:SpRY, pV1393:SpRY, and pV1524:SpRY. All subsequent experiments were performed with pV1093:SpRY. pV1093:SpRY, pV1393:SpRY, and pV1524:SpRY are available through Addgene.

### Transformation of C. albicans.

Guide RNA primers targeting alternative and classic PAM sites in the 5′ region of C. albicans *ADE2* were cloned into pV1093 and pV1093:SpRY (8). Vectors were linearized by digestion with KpnI and SacI. Repair templates to introduce premature stop codons into *ADE2* were synthesized by primer extension (8). Linearized vector, with or without purified repair template, were transformed into C. albicans SC5314 using lithium acetate transformation (15). Cells were plated on yeast extract-peptone-dextrose (YPD) supplemented with 0.27 mM uridine, 1 μg/ml adenine, and 200 μg/ml nourseothricin at 30°C. Plates were imaged after 7 days.

**FIG 1:**
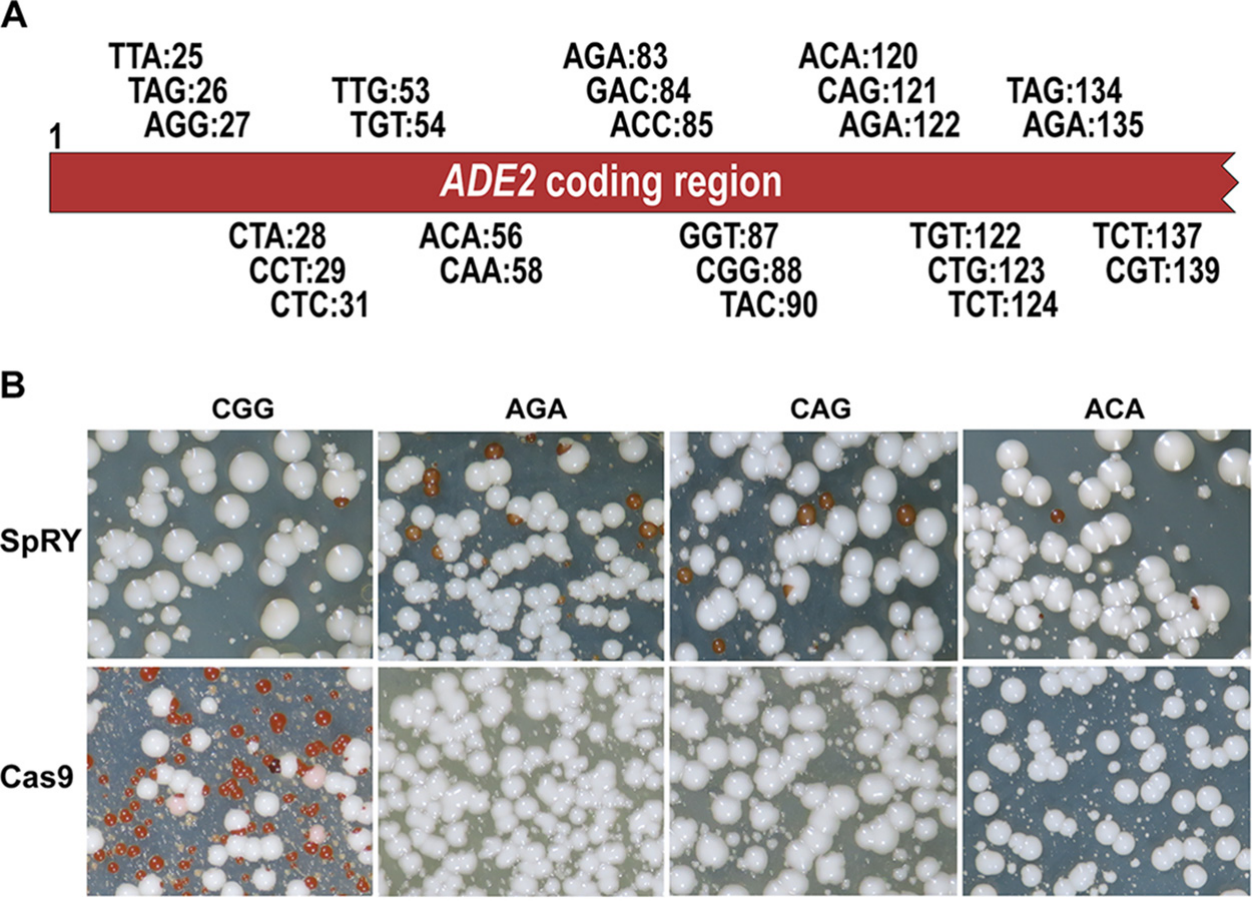
(A) PAM sites targeted between bases 1 and 150 of *ADE2*. The number after the colon indicates the first nucleotide of the PAM site as measured from the 5′ end of the *ADE2* coding region. (B) Mutation of C. albicans by CRISPR/SpRY and CRISPR/Cas9 at NGG and non-NGG PAM sequences.

We wanted to test whether pV1093:SpRY could target a variety of PAM sequences (Fig. 1A), and as such, we selected 26 trinucleotide sites spread over a 100-nucleotide stretch of *ADE2*’s 5′ end. We cloned 26 guides targeting the sequences directly upstream of these trinucleotide sites into pV1093:SpRY (Table 1). Five distinct repair templates encoding premature stop codons and with sufficient homology flanking the intended site of mutation (see Tables S1 and S2 in the supplemental material) were generated and cotransformed with the vectors. First, we tested whether pV1093:SpRY could utilize NGG PAM sites. We tested two NGG PAM sites and found that pV1093:SpRY was able to successfully edit the DNA, producing red colonies at one of these sites (Table 1). pV1093:Cas9 was able to edit at both sites and was more efficient than pV1093:SpRY at introducing mutations. Next, we tested whether pV1093:SpRY could target non-NGG PAM sites. Transformation with 9 of the 13 NRN-targeting guides and 9 of the 11 NYN-targeting guides tested resulted in successful editing (Table 1 and Fig. 1B). Wild-type Cas9 could not utilize any of the alternative PAM sites (Fig. 1B). Transformation of pV1093:SpRY without repair templates did not result in any red colonies, indicating that repair template is required for genome editing with pV1093:SpRY. Together, these data indicate that pV1093:SpRY can efficiently edit the C. albicans genome at non-NGG PAM sites.

**TABLE 1:** *ADE2* PAM sites targeted by SpRY^*a*^

PAM sequence	% red colonies	Coding noncoding strand
AGA:83	40	Coding
GAC:84	25	Coding
CAG:121	20	Coding
AGA:122	5	Coding
ACA:120	2	Coding
ACC:85	2	Coding
TAG:134	2	Coding
CAA:58	1	Noncoding
GGT:87	<1	Noncoding
CGG:88	<1	Noncoding
TAC:90	<1	Noncoding
AGA:135	<1	Coding
AGG:27	0	Coding
TAG:26	0	Coding
TTA:25	0	Coding
TGT:54	0	Coding
TTG:53	0	Coding
CTC:31	0	Noncoding
CCT:29	0	Noncoding
CTA:28	0	Noncoding
ACA:56	0	Noncoding
TGT:122	0	Noncoding
CTG:123	0	Noncoding
TCT:124	0	Noncoding
CGT:139	0	Noncoding
TCT:137	0	Noncoding

a PAM sites targeted with pV1093:SpRY. Numbers next to the PAM sequences represent the first nucleotide of the sequence as measured from the 5′ end of *ADE2*. All transformations were performed in duplicate except for AGA:83, GAC:84, and CAG:121, which were performed in triplicate. Editing efficiencies are represented as an average percentage of red colonies found across replicates.

10.1128/mSphere.00303-21.1 TABLE S1 Oligonucleotides used to target and edit *ADE2.* Guide sequences are capitalized, while lowercase letters indicate sticky ends used for cloning. For repair templates, *ADE2* sequences are capitalized and bases that will change the genome sequence are lowercase. Download Table S1, DOCX file, 0.01 MB. Copyright © 2021 Evans and Bernstein. 2021 Evans and Bernstein. https://creativecommons.org/licenses/by/4.0/ This content is distributed under the terms of the Creative Commons Attribution 4.0 International license.

10.1128/mSphere.00303-21.2 TABLE S2 List of guides and corresponding repair templates used for editing. Download Table S2, DOCX file, 0.01 MB. Copyright © 2021 Evans and Bernstein. 2021 Evans and Bernstein. https://creativecommons.org/licenses/by/4.0/ This content is distributed under the terms of the Creative Commons Attribution 4.0 International license.

Editing efficiency was highly variable among PAM sites as the frequency of successful editing ranged from less than 1% up to 40%. Furthermore, identical trinucleotide sequences, found at distinct genomic locations, displayed different editing efficiencies. For example, transformation with guides targeting TAG at base 134 produced red colonies, whereas a TAG at base 26 did not. In addition, the mutation efficiencies at three distinct AGA PAM sites were <1, 5, and 40% (Table 1). These data suggest that editing with pV1093:SpRY is dependent on more than the trinucleotide sequence targeted. As such, trinucleotide sequences we were unable to target in *ADE2* may be targetable in other genomic contexts. For example, nucleotides adjacent to the targeted trinucleotide sequence may be important for recognition by SpRY. Additionally, the presence of nucleosomes could inhibit SpRY activity at some sequences.

The variability in editing efficiency at different PAM sites is similar to previous studies which have utilized SpRY. Walton et al. used their system to edit human DNA and found the editing efficiencies to range from 20% to 90% (14). In addition, Xu et al. recently developed a CRISPR/SpRY system for rice and found editing efficiencies range from around 2% to 75% (16). In each system, NRN sequences were targeted with greater affinity than NYN sequences. Further modification of CRISPR/SpRY may lead to more uniform targeting of alternative PAM site sequences.

Here, we describe a CRISPR/SpRY system for C. albicans which does not require an NGG PAM sequence. The C. albicans genome is approximately 33% G/C (17). Traditional Cas9 editing can target 6,341 of 6,466 C. albicans genes at least once (8). The additional PAM site flexibility in our CRISPR/SpRY system has the potential to target genes that traditional Cas9 cannot target. Although off-target effects may be a concern during genome editing, these have not been extensively characterized with CRISPR/SpRY. High-fidelity variants of SpRY have been described which reduce off-target editing in human cells (14). In our experience, editing in C. albicans requires a complementary guide as well as repair template and while we have not extensively tested for off-target effects, changes to guides or the homologous ends of repair templates inhibit genome editing. CRISPR/SpRY will enable investigators to target previously untargetable DNA lacking NGG PAM sites. Such specificity and flexibility can be critical when mutation of a specific residue or region of the genome is required by the investigator.
