# Supplementary material for: SpRY Cas9 Can Utilize a Variety of Protospacer Adjacent Motif Site Sequences To Edit the Candida albicans Genome
Source: mSphere. 2021 May 19;6(3):e00303-21. doi: 10.1128/mSphere.00303-21 (PMC8265644; doi:10.1128/mSphere.00303-21)
Supplement: TABLE S1 [file msphere.00303-21-st001.docx]

**Table S1. Oligonucleotides used in this study**

| Name | Sequence |
| --- | --- |
| Guide_TTA:25_Fwd | atttgATAGCAAAACTGTTGGTATTg |
| Guide_TTA:25_Rv | aaaacAATACCAACAGTTTTGCTATc |
| Guide_TAG:26_Fwd | atttgTAGCAAAACTGTTGGTATTTg |
| Guide_TAG:26_Rv | aaaacAAATACCAACAGTTTTGCTAc |
| Guide_AGG:27_Fwd | atttgAGCAAAACTGTTGGTATTTTg |
| Guide_AGG:27_Rv | aaaacAAAATACCAACAGTTTTGCTc |
| Guide_CTA:28_Fwd | atttgACGACCTAATTGGCCACCTCg |
| Guide_CTA:28_Rv | aaaacGAGGTGGCCAATTAGGTCGTc |
| Guide_CCT:29_Fwd | atttgTACGACCTAATTGGCCACCTg |
| Guide_CCT:29_Rv | aaaacAGGTGGCCAATTAGGTCGTAc |
| Guide_CTC:31_Fwd | atttgCATACGACCTAATTGGCCACg |
| Guide_CTC:31_Rv | aaaacGTGGCCAATTAGGTCGTATGc |
| Guide_TTG:53_Fwd | atttgTGGCCAATTAGGTCGTATGAg |
| Guide_TTG:53_Rv | aaaacTCATACGACCTAATTGGCCAc |
| Guide_TGT:54_Fwd | atttgGGCCAATTAGGTCGTATGATg |
| Guide_TGT:54_Rv | aaaacATCATACGACCTAATTGGCCc |
| Guide_ACA:56_Fwd | atttgTCAATCTATGTGCTGCTTCAg |
| Guide_ACA:56_Rv | aaaacTGAAGCAGCACATAGATTGAc |
| Guide_CAA:58_Fwd | atttgATTCAATCTATGTGCTGCTTg |
| Guide_CAA:58_Rv | aaaacAAGCAGCACATAGATTGAATc |
| Guide_AGA:83_Fwd | atttgAGCACATAGATTGAATATCAg |
| Guide_AGA:83_Rv | aaaacTGATATTCAATCTATGTGCTc |
| Guide_GAC:84_Fwd | atttgGCACATAGATTGAATATCAAg |
| Guide_GAC:84_Rv | aaaacTTGATATTCAATCTATGTGCc |
| Guide_ACC:85_Fwd | atttgCACATAGATTGAATATCAAGg |
| Guide_ACC:85_Rv | aaaacCTTGATATTCAATCTATGTGc |
| Guide_GGT:87_Fwd | atttgTTGGCTGCGTCCAAAATTACg |
| Guide_GGT:87_Rv | aaaacGTAATTTTGGACGCAGCCAAc |
| Guide_CGG:88_Fwd | atttgTTTGGCTGCGTCCAAAATTAg |
| Guide_CGG:88_Rv | aaaacTAATTTTGGACGCAGCCAAAc |
| Guide_TAC:90_Fwd | atttgGATTTGGCTGCGTCCAAAATg |
| Guide_TAC:90_Rv | aaaacATTTTGGACGCAGCCAAATCc |
| Guide_ACA:120_Fwd | atttgGCAGCCAAATCACCAGCTAAg |
| Guide_ACA:120_Rv | aaaacTTAGCTGGTGATTTGGCTGCc |
| Guide_CAG:121_Fwd | atttgCAGCCAAATCACCAGCTAAAg |
| Guide_CAG:121_Rv | aaaacTTTAGCTGGTGATTTGGCTGc |
| Guide_AGA:122_Fwd | atttgAGCCAAATCACCAGCTAAACg |
| Guide_AGA:122_Rv | aaaacGTTTAGCTGGTGATTTGGCTc |
| Guide_TGT:122_Fwd | atttgGATCGTCTAGAGCATTGATCg |
| Guide_TGT:122_Rv | aaaacGATCAATGCTCTAGACGATCc |
| Guide_CTG:123_Fwd | atttgTGATCGTCTAGAGCATTGATg |
| Guide_CTG:123_Rv | aaaacATCAATGCTCTAGACGATCAc |
| Guide_TCT:124_Fwd | atttgGTGATCGTCTAGAGCATTGAg |
| Guide_TCT:124_Rv | aaaacTCAATGCTCTAGACGATCACc |
| Guide_TAG:134_Fwd | atttgAGCTAAACAGATCAATGCTCg |
| Guide_TAG:134_Rv | aaaacGAGCATTGATCTGTTTAGCTc |
| Guide_AGA:135_Fwd | atttgGCTAAACAGATCAATGCTCTg |
| Guide_AGA:135_Rv | aaaacAGAGCATTGATCTGTTTAGCc |
| Guide_TCT:137_Fwd | atttgACGATCCATCAACGTGATCGg |
| Guide_TCT:137_Rv | aaaacCGATCACGTTGATGGATCGTc |
| Guide_CGT:139_Fwd | atttgGAACGATCCATCAACGTGATg |
| Guide_CGT:139_Rv | aaaacATCACGTTGATGGATCGTTCc |
| Repair_25_Fwd | TATACAAGCACTACACATAATGGATAGCAAAACTGTTGGTATctgaagcGGTGGCCAATT |
| Repair_25_Rv | GATATTCAATCTATGTGCTGCTTCAACAATCATACGACCTAATTGGCCACCgcttcagAT |
| Repair_55_Fwd | GCAAAACTGTTGGTATTTTAGGAGGTGGCCAATTAGGTCGTATGATttaattaaCAGCAC |
| Repair_55_Rv | TTGGCTGCGTCCAAAATTACGGTCTTGATATTCAATCTATGTGCTGttaattaaATCATA |
| Repair_85_Fwd | TAGGTCGTATGATTGTTGAAGCAGCACATAGATTGAATATCAAttaattaaTTTTGGACG |
| Repair_85_Rv | TCGTCTAGAGCATTGATCTGTTTAGCTGGTGATTTGGCTGCGTCCAAAAttaattaaTTG |
| Repair_121_Fwd | TATCAAGACCGTAATTTTGGACGCAGCCAAATCACCAGCTAActgaagCAATGCTCTAGA |
| Repair_121_Rv | AATCGAATCATAGTTTGTGAACGATCCATCAACGTGATCGTCTAGAGCATTGcttcagTT |
| Repair_136_Fwd | CGTAATTTTGGACGCAGCCAAATCACCAGCTAAACAGATCAATGCTCTctgaagTCACGT |
| Repair_136_Rv | TAATTTCACAATCGAATCATAGTTTGTGAACGATCCATCAACGTGActtcagAGAGCATT |
