# Supplementary material for: SpRY Cas9 Can Utilize a Variety of Protospacer Adjacent Motif Site Sequences To Edit the Candida albicans Genome
Source: mSphere. 2021 May 19;6(3):e00303-21. doi: 10.1128/mSphere.00303-21 (PMC8265644; doi:10.1128/mSphere.00303-21)
Supplement: TABLE S2 [file msphere.00303-21-st002.docx]

**Table S2. Guides with corresponding repair template**

| **Guide** | **Repair template used** |
| --- | --- |
| Guide_TTA:25 | Repair_25 |
| Guide_TAG:25 | Repair_25 |
| Guide_AGG:27 | Repair_25 |
| Guide_CTA:28 | Repair_25 |
| Guide_CCT:29 | Repair_25 |
| Guide_CTC:31 | Repair_25 |
| Guide_TTG:53 | Repair_55 |
| Guide_TGT:54 | Repair_55 |
| Guide_ACA:56 | Repair_55 |
| Guide_CAA:58 | Repair_55 |
| Guide_AGA:83 | Repair_85 |
| Guide_GAC:84 | Repair_85 |
| Guide_ACC:85 | Repair_85 |
| Guide_GGT:87 | Repair_85 |
| Guide_CGG:88 | Repair_85 |
| Guide_TAC:90 | Repair_85 |
| Guide_ACA:120 | Repair_121 |
| Guide_CAG:121 | Repair_121 |
| Guide_AGA:122 | Repair_121 |
| Guide_TGT:122 | Repair_121 |
| Guide_CTG:123 | Repair_121 |
| Guide_TCT:124 | Repair_121 |
| Guide_TAG:134 | Repair_136 |
| Guide_AGA:135 | Repair_136 |
| Guide_TCT:137 | Repair_136 |
| Guide_CGT:139 | Repair_136 |
